# Carica Papaya in Dyspeptic States

**Published:** 1893-01

**Authors:** A. J. Park

**Affiliations:** Chicago


### CARICA PAPAYA IN DYSPEPTIC STATES.

BY A. J. PARK, M. D., CHICAGO.

Dr. Woodbury* has given an extended analysis of the physiological action of carica papaya. To his results certain cases recently coming under my observation lend clinical corroboration. It has been of especial value in all states where the digestive functions are feeble, inoperative, and seriously impaired from catarrhal complications, or from any other cause, since it is most emphatically indicated when the digestive fluids are unequal to the task of converting the ingesta to a condition preparatory to assimilation.

Case I. Young lady, æt 19. Symptoms: The patient was pale, languid, and debilitated; loss of appetite; pulse feeble, compressible, and small in volume; troubled with insomnia, and extremely nervous. The food that she took was not digested—it was simply decomposed, attended by persistent and annoying eructations of gas, acid in character; she complained of a great pain in her head, distress after meals, constipation, and irregular menses. I prescribed the following:

R. Mass. hydrarg.... ........... ............gr xii
Ex. colocynth. co............................gr. vi
Ex. belladon..................................gr. iii
Ex hyoscyamus.......................... ..gr. xii
Podophyllin.................... ............gr. ii
M. ft. et div. in pil. No. xii. S : One at bed time.

Having relieved the constipation, I prescribed papoid, bismuth and strychinine as follows:

R. Papoid............. ........................gr. xv
Bismuth sub. nit ........................... " xxx
Strychnin...... ........................... .... " 1-12
M. Div. in ch. No. x. S.: Take one powder before breakfast and one before dinner. The first powder to be preceded by a coffee-cupful of hot water, taken as hot as it can be borne.

This case represents a very numerous class, which are exceedingly common, instantly recognized, and are successfully treated when papoid is the remedial agent used. In one week this patient reported herself immensely relieved. She said that after the second day the eructations ceased, the acid condition was changed, the distress in her stomach was relieved,

*Medical Standard, Vol. XII.

the sensation of fullness in her throat disappeared, her appetite improved, the insomnia gave way to restful sleep, and to use her own forcible phrase, she "had escaped from the horrors of dyspepsia and the intensified horrors of insomnia."

Case II. A gentleman of 35—a remarkably active, clear, intellectual man of tireless energy, and a great sufferer from nasal catarrh, which he had had for fifteen years; it had utterly destroyed the sense of smell; the olfactory had ceased to respond to any appeal, and the catarrhal condition had extended to the stomach and duodenum. He complained of complete loss of appetite, pain in the head, with a furred tongue, constipation, sleepless nights, aversion to exercise, owing to his physical prostration, and a general feeling of fatigue. He had considerable palpitation of the heart, and was deeply imbued with a perpetual apprehension of the recent discovery of heart failure, from which he expected to die at any moment. Having allayed his fears of heart failure, and convinced him that his palpitation was directly attributable to his indigestion, I ordered him to take a coffeecupful of water as hot as he could sip it, half an hour before breakfast and then take one pill ten minutes before.

R. Papoid.......................................dr. i
Podophyllin.....................................gr. ii
Hydrastin.......................................gr. ii
Ex. hyoscyamus..............................scruples i
M. ft. et div. in pil. No. xx. S.: Take one pill before each meal.

In addition, I prescribed hydrastin and eucalyptus an hour after each meal, and a laxative at bedtime. The hot water was continued for ten days; then I abandoned it, and placed him upon—

R. Papoid.......................................gr. xxiv
Sod. bi-carb....................................dr. ss
Bismuth sub-nit.................................dr. ss
Elix. aromat....................................f oz. i
Aq. menth. p. q. s. ad..........................f oz. ii
M. S.: Shake the bottle and give a teaspoonful before each meal.

The catarrhal condition of his stomach, nasal passages and upper bowels proved a most formidable obstacle, and sustained a very obstinate set of symptoms to combat.

He had devoted several weeks, under the professional care of an excellent specialist in laryngology, to the treatment of his catarrh—spraying and insufflation, etc.—which only afforded him temporary relief in this humid atmosphere and changing temperature. I ordered him to continue this course of treatment for six weeks, which he did, during my absence from the city. Upon my return he presented himself at my office, and reported such a radical change in his symptoms towards complete restoration of his health—which his appearance fully sustained and vindicated—that the same line of medication was adhered to for a short time longer, and within two months from the commencement of his case, he was discharged cured.

It must be remembered that he had been treated by several excellent physicians, who had in every way met every indication and symptom that presented in his case with a host of remedies—scientifically and intelligently prescribed—aided by a long list of dietary articles of the very best quality—peptonoids, pepsin, pancreatin beef extract, maltine, with its numerous and valuable additions. He had tried the grape cure, the skimmed-milk delusion, and various other good remedies for certain conditions. Within a week from commencing the administration of that wonderful remedy his symptoms changed. His headache left him; the great distress in his stomach, which tortured him for hours after meals, ceased; his tongue became perfectly clean, bowels regular, appetite excellent, complexion clear, spirits revived, and a general appearance of returning health and rejoicing.

The ruling remedy in this case was the papoid; he having been carefully and scientifically treated for months upon the old plan of remedial agents that I have named, without any perpetual relief, and changing directly to a new course of treatment, in which papoid was the chief factor, there is but one logical conclusion to arrive at as to the remedy that wrought the change.

Case III. This patient, who had complained for several years of catarrh of the bladder, following an aggravated attack of cystitis, applied to me for advice and treatment for his urinary difficulties, which he stated were constant, painful, annoying and aggravating to the last degree of endurance.

He had traveled extensively in Europe, spent several seasons at Weis-Baden, and had tried the waters at several health resorts in Germany, returning home without much improvement except of a transient character.

As the patient was over 70 years old, and greatly broken in health, I was not at all sanguine of affording him much relief, and not anxious to undertake the case, which I fully explained to him. That his age and the many years of his physical martyrdom were decidedly against his recovery, all of which he at once conceded, and stated that he only expected and only sought temporary relief. His array of symptoms were truly formidable. He was suffering from dyspepsia, with a full train of attendant manifestations; was in constant dread of heart failure, pain in epigastric regions after taking the slightest nourishment, and complained of acrid eructations and throwing off gas from the stomach; was much troubled with palpitation of the heart, with an intermittent pulse, with a feeling of a general surrender to the persistent invasion of diseases.

His vesical catarrh was a prominent feature in the multiplicity of his murmurings, and his chamber more than established the fidelity of his painful recitals.

I prescribed the following treatment for him, to relieve his costive habit and to arouse his inactive and torpid liver and secretions. I ordered:

R. Mass. hydrarg.............................gr. xxiv
Ex. colocynth, co.............................gr. xii
Podophyllin.....................................gr. iv.
Leptandrin.......................................gr. vi
Ex. hyoscyamus...............................gr. xxiv
Ex belladonna...................................gr vi
Ex. nux vomica.................................gr. vi
M. ft. et div. in pil. No. xxiv. S.: Take one pill at bedtime

After five days I commenced the following plan: I washed his bladder out three times a week; first with equal parts of milk and water, pretty warm, then with boric acid, and lastly, with hydrastin and papoid.

I gave him papoid in combination with soda bicarb. and bismuth, and ordered him to take six ounces of water as hot as he could, every morning, with ten grains of sodium chloride, and to take the papoid mixture in the middle of his meals; and once a week he was subjected to vigorous massage by a strong and healthy operator.

I regulated his diet to conform to the indications of treatment. In six weeks he had gained five pounds in weight; the mucus that formerly threatened supremacy in his chamber had almost disappeared His digestion was immensely improved, his appetite had returned, and to use his own words: "That papoid is surely the long-sought rejuvenating elixir of youth." I gave papoid the credit, because he had traveled the old line of treatment through many years of patient, preserving and unfailing faith, suffering the pains and penalties attending misdirected effort and stubborn adherence to remedies which have survived scientific application. When the secretions of the stomach are in perfect accord with those remedies the results are quite satisfactory, but administration of pancreatin, pepsin and pepsin mixtures indiscriminately, without regard to the condition of the stomach, is a blind and empirical method of meeting the abnormal condition presented.

